# Novel Anthropometric Indices and Probability of Adequate Nutrient Intake in the Older Polish Population

**DOI:** 10.3390/nu17233666

**Published:** 2025-11-24

**Authors:** Agata Białecka-Dębek, Elżbieta Wierzbicka, Olga Januszko, Barbara Pietruszka, Aleksandra Szybalska, Małgorzata Mossakowska

**Affiliations:** 1 Department of Human Nutrition, Institute of Human Nutrition Sciences, Warsaw University of Life Sciences, 02-776 Warsaw, Poland; elzbieta_wierzbicka@sggw.edu.pl (E.W.);; 2 International Institute of Molecular and Cell Biology, 02-109 Warsaw, Poland; 3 Polish Society of Gerontology, Mazovia Branch, 01-826 Warsaw, Poland; 4 Division of Preventive Medicine and Education, Department of Hypertension and Diabetology, Medical University of Gdansk, 80-214 Gdansk, Poland

**Keywords:** anthropometric indices, body roundness index, micronutrient intake, older adults, cross-sectional study

## Abstract

**Background**: The Body Roundness Index (BRI) is an emerging anthropometric measure that reflects central adiposity, particularly relevant in ageing populations where body composition and nutritional status undergo significant changes. This study aims to explore patterns linking BRI values, age, energy intake, and micronutrient adequacy using cluster analysis, with a focus on implications for older adults. **Methods**: Data from 1504 community-dwelling older adults (mean age 74.4 ± 10.8 years) in Poland participating in the PolSenior project were analyzed. K-means cluster analysis was applied to standardized variables (BRI, age, energy intake, and micronutrient adequacy) to identify major participant profiles. **Results**: The data indicate that older adults, particularly those over 75 years old, are at an elevated risk of inadequate micronutrient intake, especially for essential nutrients such as calcium, magnesium, folate and vitamin D. Three distinct clusters were identified: Cluster 1 (n = 495, 33%): oldest participants, lowest BRI, and insufficient energy and micronutrient intake, indicating a high risk of undernutrition; Cluster 2 (n = 557, 37%): average age, moderate BRI, and highest energy and micronutrient intake, suggesting a potentially excessive energy balance. Cluster 3 (n = 452, 30%): the youngest group with the highest BRI and the lowest energy and micronutrient intake, indicating early-onset central adiposity and poor dietary quality. **Conclusions**: Three clusters were identified that differed significantly in BRI, age, and adequacy of energy and micronutrient intake. BRI combined with dietary indicators effectively distinguishes nutritional risk profiles among older adults. A low BRI may indicate a risk of undernutrition in advanced age, whereas a higher BRI with low nutrient adequacy suggests poor diet quality, even within the older population. Age-specific and nutrition-sensitive interventions are needed to support healthy ageing.

## 1. Introduction

Maintaining a nutrient-dense diet, regardless of age, is a fundamental condition for maintaining both physical and mental health. Adequate dietary interventions may help postpone or prevent age-related chronic illnesses, such as cardiovascular or neurological diseases, hypertension, and diabetes mellitus [1,2,3]. During ageing, energy requirements gradually decline, while the need for vitamins and minerals remains largely unchanged, making nutrient density a key determinant of diet quality [4,5]. Aging is associated with a reduced appetite and an average annual decline of 0.5% in energy intake, which may lead to undernutrition in vulnerable older adults [6,7]. Undernutrition is frequently reported in the literature as a common phenomenon among older adults, yet it is often poorly recognized and underdiagnosed [8]. The problem of malnutrition, along with nutrient deficiencies, affects the elderly in various countries, exacerbating existing health issues and significantly deteriorating the quality of life in old age [9].

Paradoxically, older adults are also at risk for overnutrition, leading to overweight and obesity, which can exacerbate chronic conditions. Epidemiological studies indicate that the percentage of overweight and obese adults increases with age [10]. The body mass index (BMI) is widely used to classify individuals as being overweight or obese. However, many authors emphasize that BMI alone does not accurately reflect body composition or the distribution of fat tissue, which is crucial for assessing health risks in older adults [11,12,13]. Abdominal obesity, characterized by excessive accumulation of fat in the abdominal cavity, leading to increased waist circumference, significantly increases the risk of cardiovascular disease [14]. Abdominal obesity is unfavorable because it is a significant risk factor for systemic inflammation, insulin resistance, and metabolic syndrome [15]. Waist circumference and hip circumference are often used to compensate for the limitations of BMI in accurately reflecting the distribution of body fat. However, these measurements cannot account for the larger waist or hip circumference in taller individuals [16]. Thomas et al. [17] introduced the Body Roundness Index (BRI) as a parameter for estimating visceral adiposity and overall body fat. Subsequently, BRI has been recognized as a novel anthropometric index for quantifying visceral fat and related health risks [17].

In older adults, BRI reflects abdominal obesity, is associated with visceral adipose tissue and markers of inflammatory risk, and may be related to sarcopenic obesity [18]. Recent research demonstrated that BRI can be applied in elderly populations to assess risks for various health outcomes, including frailty [19], depression [20], cognitive impairment [21], and cardiovascular disease [22]. Despite these findings, the relationship between BRI and dietary adequacy among older adults remains insufficiently explored.

In this study, we examined how BRI relates to age, energy intake, and the probability of adequate micronutrient intake—defined as the extent to which an individual’s dietary intake meets established dietary reference values for essential vitamins and minerals. This adequacy was based on a probabilistic estimate using standardized Z-scores for micronutrient intake, reflecting the probability that nutrient requirements are met. Our analysis aims to identify patterns that may inform targeted nutritional strategies for ageing populations. There is a lack of current data on dietary intake in large, representative research groups of older adults and their relationship with anthropometric parameters.

## 2. Materials and Methods

### 2.1. Study Design and Participant Recruitment

The study was part of PolSenior, a cross-sectional, multidisciplinary, population-based study conducted on a nationally representative, noninstitutionalized sample of Polish Caucasian seniors between 2008 and 2011. The study protocol was approved by the Bioethics Committee of The Medical University of Silesia (KNW/0022/KB1/38/II/08/10; KNW-6501-38/I/08). Each respondent signed a written informed consent form. Briefly, all older adults were visited at home, where data were collected through face-to-face interviews. The total cohort consisted of 5695 community-dwelling participants (2899 males and 2796 females). All respondents were required to be mentally and physically capable of participating in the study protocol. Information related to sociodemographic, behavioral, or lifestyle characteristics, physical activity level, and self-rated health status was collected during the interviewer-administered questionnaire in the participant’s home visits. Detailed study design, methods, and cohort selection data, as well as measurement protocols, have been described elsewhere [23]. In brief, sociodemographic characteristics (age, education, marital status, and place of residence) were obtained using a structured questionnaire. Physical activity level was classified as low, moderate, or high based on self-reported frequency and intensity of exercise. At the same time, self-rated health status was evaluated using a numerical scale ranging from 0 (indicating the poorest health status) to 10 (indicating the best possible health status) [23].

The food-recorded questionnaires were submitted to the Department of Human Nutrition of the Warsaw University of Life Sciences by the coordinating center, exclusively for participants for whom dietary examination was performed—a total of 2046 older adults. Participants were excluded if they had serious errors in their three-day food records (n = 514) or lacked data for all anthropometric measurements (n = 28). Ultimately, 1532 participants were included in the analyses of adequate nutrient intake, and 1504 participants were included in the cluster analyses (Figure S1). The mean age was 74.4 ± 10.8 years (range: 55–102 years). The characteristics of the participants are presented in Table S1, categorized by sex.

### 2.2. Data Collection

Data on dietary intake were assessed using 3-day food records conducted on consecutive days, including one weekend day [24,25]. To obtain accurate data, participants and trained interviewers collected information about the types of foods, preparation methods, ingredients in dishes and recipes, and, if possible, the brand names of products. The amounts of each food could be estimated using household measures and a photographic album denoting the actual serving sizes of foodstuffs and related products. Household measures reported in the food records were detailed into weighted amounts. Questionnaires were analysed and transformed into energy and nutrient intake data using the corresponding ‘Dieta 5D’ software (National Food and Nutrition Institute, Warsaw, Poland), based on available data in the Polish food composition table [26]. To account for potential misreporting, unreliable or incomplete energy intake was defined as values (<800 or >4200 kcal/d for men and <500 or >3500 kcal/d/for women) [27]. However, no participants had intakes outside of these ranges. Results for micronutrients were based solely on dietary intake data, excluding supplements and other sources. Energy and nutrient intakes were calculated individually for each participant, and the resulting intakes were compared with age- and sex-specific reference values to assess adequacy. The average of the three days of dietary intake data was used for analysis. In 2024, the national Dietary Reference Intakes were revised. From that point onwards, adequacy assessment in the study was aligned with the updated reference values [28].

### 2.3. Dietary Assessment

For vitamins and minerals intake, the statistical method of probability (the z-score value) was used to assess the adequacy of nutrient intake [24,25], based on the Estimated Average Requirement (EAR), and in the case of vitamins D and E, based on the Adequate Intake (AI). The EAR is the mean daily intake value estimated to meet the requirements of half of the healthy individuals in a life-stage and sex group and to prevent chronic diseases. The AI is derived when there is insufficient evidence to establish an EAR. For vitamin D and E intake, over 100% of the AI was estimated as adequate. The z-scores were calculated using ‘Dieta 5D’ software (National Food and Nutrition Institute, Warsaw, Poland). The z-scores were calculated according to the following equation [25]:

$$z-score=\frac{D}{SD_{D}}=\frac{y-EAR}{\sqrt{{({SD}_{R}}^{2}+\left(\frac{{SDv}^{2}}{n}\right))}}$$

where D—difference between observed individual intake/consumption (y) and the EAR value; SD_D_—standard deviation of D; y—observed average individual intake; SD_R_—standard deviation of the requirements (estimated as 10, 15 and 20% of the EAR according to the nutrient; 10% for calcium, zinc, magnesium, iron, phosphorus and vitamins: C, thiamine, riboflavin, B_6_, B_12_ and folate; 15% for copper and niacin, 20% for iodine and vitamin A); SD_V_—standard deviation of intake in the study group; n—number of days that intake was assessed.

The use of the z-score indicator enables qualitative inference, based on a quantitative analysis, and thus:

- if D/SD_D_ is greater than 1, then there is a lot of confidence that the usual intake of a nutrient is adequate,
- if D/SD_D_ is lower than −1, then it is certain that the usual intake of a nutrient for the analyzed person is inadequate,
- if D/SD_D_ is between −1 and 1, then it cannot be determined with certainty whether the intake of an individual is adequate or inadequate [25].

For this study, adherence to recommended vitamin and mineral intakes was estimated for each nutrient and summarized as a composite Micronutrients Score (MS), ranging from 0 to 17 points. The scoring approach was based on the standardized z-score (D/SD_D_): 1 point for D/SD_D_ > 1; 0.5 points for −1 ≤ D/SD_D_ ≤ 1; and 0 points for D/SD_D_ < −1. Higher MS values denote greater compliance with recommended micronutrient intakes among participants.

### 2.4. Anthropometric Variables

During the home visit, participants were weighed, wearing light clothing and without shoes, using a portable digital scale. Their height was measured with a stadiometer under standardized conditions. Waist circumference (WC) was determined by using an anthropometric tape at the midpoint between the lowest rib and the iliac crest. Details of measurement protocols have been described elsewhere [23].

Body mass index (BMI) was calculated as weight (W) divided by the square of height (H) expressed in meters (kg/m^2^). BMI < 25 kg/m^2^ was considered normal, 25 kg/m^2^ ≤ BMI < 29.9 kg/m^2^ was considered overweight, and BMI ≥ 30 kg/m^2^ was considered obese [29]. Additionally, indices of abdominal obesity, including the waist-to-hip ratio (WHR) and the waist-to-height ratio (WHtR), were calculated. WC values of ≥80 cm in women and ≥94 cm in men were considered indicative of an increased risk of obesity-related diseases, while values of ≥88 cm in women and ≥102 cm in men denoted a high risk [30,31]. WHR was classified as ≥0.90 for men and ≥0.85 for women, indicating increased abdominal obesity [29], whereas WHtR was considered as 0.5 for both sexes, denoting elevated cardiometabolic risk [32,33].

In the present study, novel anthropometric indices related to both total body fat and abdominal adiposity were examined. Among these, the Body Roundness Index (BRI) was applied in this study, with values calculated using the following formula:

$$BRI=364.2-363.5\sqrt{1-(\frac{{\left[\frac{WC(m)}{2\pi }\right]}^{2}}{{\left[0.5H\left(m\right)\right]}^{2}})}$$

where WC—waist circumference (m); H—height (m); π—mathematical constant (circumference to diameter ratio) [17].

### 2.5. Statistical Analysis

According to the results, continuous variables are shown as the mean and standard deviation (SD), whereas categorical variables are expressed as frequencies (n) and percentages (%). The Kolmogorov–Smirnov test was performed to check the normality of the data distribution. Differences between subgroups for continuous variables were tested using one-way analysis of variance (ANOVA) for normally distributed data, and the Mann–Whitney U test or the Kruskal–Wallis test in cases where the data were not normally distributed. Differences between groups were determined using Bonferroni post hoc analyses. For categorical variables, the Chi-squared test was applied with two-tailed *p*-values.

A cluster analysis was performed to identify the BRI patterns. The K-means algorithm was applied to the BRI values of Polish older adults to identify similarity clusters/subgroups within this population, taking into account age as well as energy and micronutrient intake (expressed as a score relative to age-specific dietary reference intakes). All input variables were standardized and expressed as Z-scores. The number of clusters was determined using the Elbow method, which evaluated the within-cluster sum of squares and the shape of the curve on the plot. The optimal number of clusters was selected based on the point at which adding another cluster did not significantly improve model performance. Based on this, three clusters were identified.

All statistical analyses were carried out using the STATISTICA 13.3 software (TIBCO Software Inc., Palo Alto, CA, USA), taking *p*-value < 0.05 as the statistical significance threshold.

## 3. Results

### 3.1. Probability of Adequate Nutrient Intake

Table 1 presents the average daily intake of energy and macronutrients by sex and age group. The average daily energy intake was statistically significantly higher in men (1761 ± 518 kcal) when compared to women (1494 ± 433 kcal). Consequently, for most nutrients, the average daily intake was significantly lower in women compared to men, except for vitamin A, for which the differences were not statistically significant (Table 2 and Table 3).

The average intake of energy and nutrients was compared across age groups: 55–65 years, 66–75 years, and above 75 years. For most nutrients, the average intake was lowest in the oldest group (>75 years) compared to the youngest groups (Table 1 and Table 4).

Table 2 shows the average daily intake and adequacy of vitamin and mineral intake, assessed by z-scores. The highest proportions of people with inadequate intake were observed for folic acid (76.9% of men and 77.2% of women), calcium (83.3% of men and 92.3% of women), and magnesium in men (65.6%). Furthermore, less than one-third of the study population had an adequate intake of vitamin A, vitamin C, thiamine, vitamin B_12_, and zinc. Inadequate intakes of vitamin C, thiamine, riboflavin, magnesium, and zinc were more common in men than in women, and inadequate intakes of niacin, vitamin B_6_, calcium, phosphorus, iron, copper, and iodine were more common in women than in men.

Only a very small percentage (0.6% for men and 0.3% for women) covers the recommendations for vitamin D intake. For vitamin E, adequate intake was observed in 20.9% of men and 32.5% of women (Table 3).

Table 4 presents the average daily dietary intake and adequacy of intake for 15 micronutrients across three age groups. For most vitamins and minerals, there was a statistically significant decline in adequate intake and a corresponding increase in inadequate intake as age advanced. Two micronutrients stand out for their extremely high rates of inadequacy across all age groups. Calcium intake was alarming, deficiency affecting 77.7% of the 55–65 group, rising sharply to 91.2% in the 66–75 group, and remaining very high at 89.0% in the >75 group. Additionally, folate intake was inadequate in a large majority of individuals, ranging from 73.5% (55–65 years) to 79.4% (>75 years). There were no statistically significant differences between age groups in terms of adequate intake of vitamin A and B_12_.

The adequacy of micronutrient intake and anthropometric indices (BRI, BMI, WC, WHR, WHtR) was analyzed separately in men (Table S2) and women (Table S3). In the case of men, it was observed that the group with insufficient iron intake had significantly lower BRI, BMI, WC, and WHtR values compared to the groups with adequate or indeterminate intake of other micronutrients. For the remaining micronutrients, no statistically significant differences were noted (Table S2).

In the case of women, groups with adequate intake of folates and magnesium had statistically significantly lower BRI, WHR, and WHtR values compared to groups with insufficient or indeterminate intake. Additionally, the group with adequate intake of vitamin C and zinc had significantly higher BMI values. On the other hand, the group with adequate intake of vitamin C and iron had significantly lower WHR values (Table S3).

### 3.2. BRI Patterns and General Characteristics by Clusters

**Figure 1:**
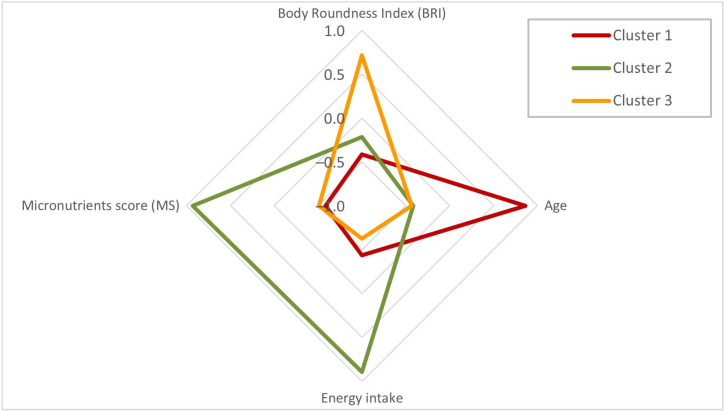
BRI patterns by cluster analysis according to the input variables (Z-scores) among older adults (n = 1504). The figure shows the patterns of Body Roundness Index (BRI), age, energy intake, and micronutrient score (MS) across three clusters of older adults. Values are presented as standardized Z-scores. Cluster 1 (red) is characterized by low BRI, low energy intake, and higher age; Cluster 2 (green) shows higher energy intake, micronutrient score, and low BRI; Cluster 3 (orange) exhibits higher BRI with low energy intake, and micronutrient score.

Figure 1 shows the standardized Z-scores of BRI, age, energy intake, and micronutrients score value across three clusters: Cluster 1 (n = 495), Cluster 2 (n = 557), and Cluster 3 (n = 452).

In terms of BRI (Figure 1 and Table S4), Cluster 3 (30% of participants) demonstrated the highest BRI (Z-score = 0.72), indicating greater central adiposity compared to Cluster 1 (Z-score = −0.41) and Cluster 2 (Z-score = −0.21). The differences in BRI between Cluster 1 and both Cluster 2 and Cluster 3 were statistically significant (*p* < 0.05), highlighting a gradient of increasing adiposity from older to younger groups.

In contrast, Cluster 1 (33%) was characterized by the oldest participants (Z-score = 0.86), significantly older than those in Cluster 2 (−0.41) and Cluster 3 (−0.43) (*p* < 0.05). No significant age difference was observed between Clusters 2 and 3, suggesting that the primary distinction in age occurred between Cluster 1 and the two younger clusters.

Regarding nutritional variables, Cluster 2 (37%) exhibited the highest intake of both energy (Z-score = 0.90) and micronutrients (Z-score = 0.93). These values were significantly greater than those observed in Cluster 1 (energy = −0.44; micronutrients = −0.58) and Cluster 3 (energy = −0.62; micronutrients = −0.51). The differences between Cluster 1 and Cluster 2, as well as between Cluster 2 and Cluster 3, were statistically significant for both dietary variables (*p* < 0.05). In contrast, the comparison between Clusters 1 and 3 was not statistically significant (Table S4).

### 3.3. BRI Patterns, Age, Energy Intake, and Micronutrients Score by Clusters

Table 5 shows the results of the BRI values stratified by clusters. Statistically significant differences were observed. Cluster 3 presented the highest BRI (7.08 ± 1.96), significantly greater than both Cluster 2 (5.34 ± 1.57) and Cluster 1 (4.97 ± 1.37), while Cluster 1 included the oldest participants (83.6 ± 7.5 years). Cluster 2 showed the highest energy intake (2076 ± 441 kcal) and micronutrients score (11.5 ± 1.6), indicating a more adequate micronutrient intake compared to Clusters 1 and 3, which showed similarly lower intakes despite differing BRI values.

### 3.4. BRI Patterns, Socio-Demographic, Physical Activity Level, and Self-Rated Health Status by Clusters

Cluster analysis identified three statistically significant subgroups, differing in terms of socio-demographics, physical activity levels, and self-rated health status (Table 6). Cluster 1 represented the oldest group (mean age: 84 years), with 82% aged over 75, and included a slightly higher proportion of men (55%). 55% participants were widowed or divorced, and only 9% had higher education, indicating lower socioeconomic status. This group exhibited the lowest mean BRI and the lowest intake of energy and micronutrients. Notably, a substantial percentage (33%) reported low levels of physical activity, and only 23% rated their health status in the highest range (8–10 points) (Table 6).

In contrast, Cluster 2 was characterized by a mean age of approximately 70 years, with the most significant proportion in the 66–75 age group (42%). Participants were more often married (66%), more highly educated (17% with higher education), and reported the highest intake of energy and micronutrients. Physical activity was generally high (only 12% reported low levels), and 29% rated their health status as high (8–10 points). Despite their moderately elevated BRI, this group appears to maintain an overall favorable nutritional and functional status. However, the elevated BRI and high energy intake could signal a positive energy balance, which, if physical activity declines, raises the risk of adiposity with advancing age (Table 6).

Cluster 3 included participants with a similar mean age to Cluster 2, but a markedly different profile. This group was predominantly female (60%), had lower education levels, and a lower proportion of participants were married (59%). Despite a low intake of energy and micronutrients, Cluster 3 exhibited the highest BRI, indicating central adiposity unrelated to excess energy and micronutrient intakes, which may be potentially driven by poor diet quality and low physical activity level. Self-rated health was modest (only 22% reported as high) (Table 6).

## 4. Discussion

This study aimed to estimate the probability of adequate nutrient intake in the context of anthropometric indices, notably the Body Roundness Index (BRI), a relatively new indicator, in a population of older Polish adults, thereby filling a gap in current data on the association between diet and anthropometric parameters in this group. The findings provide a comprehensive overview in this population, revealing substantial heterogeneity across age and sex groups. Both energy and micronutrient intakes tended to decline with advancing age, with women generally at a higher risk of inadequate intakes compared to men. Several micronutrients—particularly, calcium, magnesium, vitamin D, and folate—were consumed below recommended levels by a considerable proportion of participants. The cluster analysis identified three distinct profiles, indicating that differences in nutritional status, as assessed by anthropometric measures, were associated with variations in dietary adequacy.

Research indicates that BRI is a more accurate reflection of health risk than BMI [34]. Recent publications confirm that BRI is a stronger predictor of adverse health outcomes than BMI, due to its ability to estimate visceral fat distribution better [17]. A study by Pratt et al. demonstrated that BRI correlated better with body composition measured by DXA for android to gynoid fat mass and fat mass index than BMI [35]. Additionally, BRI demonstrated higher predictive value for metabolic conditions such as nonalcoholic fatty liver disease (NAFLD) [36]. High BRI is strongly associated with an increased risk of frailty, with BRI being a better predictor of frailty than BMI [37]. Furthermore, elevated BRI is associated with an increased risk of incident psychiatric disorders, including depression and anxiety, in older adults [38]. Low BRI values in older adults may also have important clinical implications, as they can reflect reduced muscle mass and insufficient fat reserves, both of which are characteristic of early sarcopenia or frailty. Therefore, BRI should be considered not only as a marker of central adiposity but also as a potential indicator of underweight or muscle depletion [19].

Our study reveals that, similar to other older populations, Poland exhibits a high prevalence of inadequate intake of key micronutrients, including iron, folate, magnesium, calcium, and vitamin D [39]. Our analysis revealed diverse and sex-specific relationships between anthropometric indicators and micronutrient adequacy.

In men, the group with insufficient iron intake was found to have significantly lower BRI, BMI, WC, and WHtR values. This observation, combining low obesity rates with nutrient deficiency, is alarming and may signal wasting or undernutrition, which is a predictor of poorer prognosis in seniors [40]. Lower anthropometric indicators in this group may reflect poor nutritional status, with BMI and body weight being the best predictors of hemoglobin levels and thus, indirectly, iron status [41]. Further research is needed to determine whether low BRI in older men can serve as an early predictor of the risk of nutritional deficiencies, including iron.

A more complex picture was observed in women. Groups with adequate intakes of magnesium and folate had significantly lower BRI, WHR, and WHtR values. Magnesium deficiency is particularly problematic, as it is common [39]. In our study, the percentage of people with a probability of insufficient intake was high (66% for men and 33% for women). The results of the national Polish study (WOBASZ) [42] also indicated an insufficient intake of magnesium in older adults. Similar results apply to other European studies [43], including the ENHRII project with 25 participating countries [44]. Adequate magnesium intake has been linked to a lower risk of abdominal obesity and metabolically healthy obesity (MHO) [45]. This association is consistent with broader reports of sex differences in diet quality and their impact on BMI [46]. The association between higher magnesium intake and lower adiposity and insulin resistance is well documented, also in the context of preventing metabolic syndrome in older adults [47]. In addition, studies have shown an association between higher magnesium intake and lower body fat, including in individuals with impaired glucose tolerance and type 2 diabetes [48,49]. Importantly, the benefits are more pronounced with dietary magnesium than with supplements, underscoring the importance of magnesium-rich foods [50]. Studies of large groups of middle-aged and elderly individuals, including those with metabolic risk factors, have shown that those with higher magnesium intake have greater skeletal muscle mass [50,51].

For folate, in our study, 77% of both men and women had a high probability of insufficient intake, while the literature reports lower inadequacy [44], and the highest proportion of women and men with intakes below the EAR was observed in the Netherlands (29% of women and 16% of men) [43]. An observational study of healthy adults found that folate intake and serum folate levels were inversely associated with body fat percentage and waist circumference. However, only serum folate levels, and not intake, were associated with BMI [52]. Some studies that did not focus on individuals aged 60 years and over have shown an association between BMI and serum folate levels [53,54]. However, this is not supported by studies of older individuals, for whom no association was found between folate levels and nutritional status or BMI [55,56]. Nevertheless, there has been no confirmation through randomized controlled trials in older individuals of significant changes in BMI or body composition over time resulting from folate supplementation, indicating that the observed associations may not be causal or may be influenced by other factors [57].

Another finding was that the groups with adequate vitamin C and zinc intake had higher BMI values, while the group with adequate vitamin C and iron intake had lower WHR values. This finding highlights the potential limitation of BMI alone in older adults, where higher BMI may, albeit inconsistently, reflect higher muscle mass or better overall nutritional status in the absence of deficiencies [58]. At the same time, the lower WHR in the group with adequate vitamin C and iron intake, despite a higher BMI, suggests that body fat distribution (as measured by WHR) may be more sensitive to diet quality.

Cluster analysis provided more profound insight into how BRI was associated with dietary and sociodemographic patterns. The identification of three statistically significant subgroups with two distinct risk profiles highlights the heterogeneity of the Polish older adult population. This finding confirms the U-shaped relationship between BRI and adverse health outcomes and mortality in the elderly population, as observed in other studies [37,59]. Cluster 3, despite low energy and micronutrient intake, was characterized by the highest BRI and a female predominance. This result is critical because it potential risk profile for “undernutrition with obesity” [60]. High BRI coexists with poor dietary quality, which threatens muscle loss and leads to frailty, with which BRI is strongly associated [37]. Cluster 1, the oldest, was characterized by the lowest BRI and the lowest energy and micronutrient intake. A low BRI, combined with low intake and the lowest physical activity index, is an indicator of wasting and/or sarcopenia [40]. This group, characterized by low socioeconomic status (low education level), was most vulnerable to malnutrition and poorer prognosis, which is consistent with Polish studies indicating that low socioeconomic status is an independent correlate of malnutrition risk [61].

Cluster 2 exhibited the highest energy and micronutrient intakes, along with moderately elevated BRI. High micronutrient intakes suggest high dietary quality, which may buffer the adverse effects of moderately elevated BRI and overall energy intake. According to the literature, an adequate intake of micronutrients, such as vitamin D and calcium, is crucial for maintaining lean body mass and preventing sarcopenia in older adults, even at higher body weights. Among the study participants, vitamin D and calcium deficiency remain a significant problem [39]. This is also emphasized by the results of other authors. In a study comparing European countries, it was highlighted that for calcium percentage with intakes below the EAR, it was exceptionally high for Poland (55% of women and 48% of men) [43]. Similar results were obtained in our study. Calcium is an essential nutrient for maintaining bone health, but the relationship between dietary calcium intake and bone mineral density in older adults remains controversial [62,63]. Several studies have found a correlation between calcium intake and body composition, particularly in older adults. Several studies have demonstrated an association between higher calcium intake and lower body fat levels [64,65], as well as a reduced risk of conditions such as osteosarcopenia [66]. However, other studies have shown no significant effect of calcium supplementation on body weight or fat mass [67].

The studies showed that the micronutrient with the highest prevalence of inadequacy in the older population was also vitamin D, which is confirmed by the results of our study. The percentages below the EAR were generally high (87–100%) [43]. The results of the WOBASZ study, which involved 1338 older people aged 60–74 from Poland, also indicated an insufficient intake of vitamin D [42]. Studies show a clear relationship between vitamin D levels and body composition in older adults. Greater dietary vitamin D intake is associated with increased lean mass, a better muscle-to-fat ratio, and lower total body weight, fat mass, and visceral fat, particularly in the context of weight loss and lifestyle interventions among older adults with metabolic syndrome [68]. Observational studies have shown that lower serum vitamin D levels are associated with a higher percentage of body fat [42], particularly in individuals with sarcopenic obesity [69]. Some evidence suggests that adequate vitamin D intake may help prevent muscle mass loss in older people with type 2 diabetes [70]. High levels of vitamin D appear to exacerbate the decline in body fat associated with physical activity during aging; however, this mechanism may be due to higher doses of moderate to intensive outdoor physical activity, rather than a direct effect of 25OHD [71]. However, large randomized controlled trials have shown that daily vitamin D3 supplementation, compared to a placebo, does not improve body weight or composition in the general elderly population [72].

The results from the SUSFANS project show that there is considerable variation in food and nutrient intakes across Europe, not only between countries, but also within them. Within countries, food intake varied by socio-economic factors, including age, sex, and educational level. Within countries, nutrient density of the diet tended to be higher for women [73]. Some studies suggest that older women appear to be the most vulnerable group across countries, with additional risk of potassium, calcium, and folate deficiency [74]. At the same time, the results of others indicate that in the adult and elderly populations, the prevalence of inadequate micronutrient intakes was higher in females than in males for all micronutrients except vitamin C [44]. The results of our study indicate that for most of the nutrients the average intake was significant lower in women compared to men, except vitamins A and C. Still, inadequate intakes were more common in women than in men only for or niacin, vitamin B_6_, calcium, phosphorus, iron, copper and iodine, and for vitamin C, thiamine, riboflavin, magnesium and zinc inadequate intakes were more common in men than in women.

### Strengths and Limitations

Our study was the first to use the BRI to assess the association between central obesity and the probability of adequate nutrient intake in a large sample of older Polish adults, using current dietary guidelines for 2024 and a probabilistic approach. These results underscore the importance of age-sensitive interpretation of anthropometric indicators such as BRI, particularly in older adults. Integrating BRI with a comprehensive dietary assessment provides a more accurate understanding of health risk than using either measure alone. This study has several notable strengths. Firstly, it involved a large cohort of older adults in Poland, enhancing the generalizability of its findings within this population. Secondly, a significant strength lies in its assessment of dietary intake adequacy against the new nutritional standards for the Polish population in 2024. The reliability of the results is strengthened by the probability method employed, which accounts for individual variability by providing a more nuanced understanding of dietary patterns. The key limitation of this study is the potential inaccuracy of the BRI as a measure for older adults. Measuring height in this demographic is often subject to error, and, crucially, the BRI primarily assesses abdominal fat rather than muscle mass. This is a significant issue because measuring muscle mass is vital for determining the risk of sarcopenic obesity, a condition characterized by the coexistence of muscle loss and excess fat, which is common in older individuals. Future studies should combine the BRI with measurements of muscle mass to more fully assess the risk of sarcopenia in the context of dietary adequacy. It is important to note that BRI thresholds have not yet been validated in older populations, and the implications of low BRI for sarcopenia or frailty in this age group warrant further investigation. Additionally, the study lacked biochemical data, which could have provided a more objective evaluation of nutritional status. The use of a 3-day dietary recall may also introduce recall bias and may not fully capture day-to-day variability in food intake. Nevertheless, standardized procedures and trained interviewers were used to improve data reliability. Another limitation is that the Micronutrient Score (MS) used in this study has not yet been validated, which may affect the precision of nutrient adequacy assessment. Several potential confounding factors may have contributed to the observed differences between clusters. Beyond age, sex, socioeconomic status, physical activity, and overall health status, additional factors such as chronic diseases common in older adults, variability in appetite and gastrointestinal function, unmeasured medication use, smoking and alcohol consumption, and seasonal variations in dietary habits may influence both anthropometric indicators and micronutrient intake. Limitations of this study include its cross-sectional nature, which prevents the establishment of a cause-and-effect relationship. Longitudinal studies are needed to clarify the temporal and causal relationships between BRI and nutritional status in older populations. Finally, the timeframe of data collection (2008–2011) may be too dated to reflect current dietary patterns. However, these data remain valuable for understanding long-term relationships between anthropometric indices and nutrient adequacy in the older population, as such associations are generally stable over time.

## 5. Conclusions

The findings from this study indicate that older adults with low BRI and inadequate energy and micronutrient intake (Cluster 1) may be at risk of undernutrition and age-related functional decline. Younger older individuals with high BRI and poor dietary intake (Cluster 3) demonstrate signs of central adiposity despite nutritional insufficiency. Middle-aged individuals with high energy and micronutrient intake and moderately elevated BRI (Cluster 2) likely reflect a state of positive energy balance, underscoring the importance of balancing diet quality and physical activity. These findings support the utility of BRI as an anthropometric indicator when considered in conjunction with dietary intake and age, particularly in older populations. The results underscore the need for age-specific and nutrition-sensitive approaches in health and nutrition, targeting the prevention of undernutrition in older people and promoting energy balance in middle-aged populations, with clear implications for nutritional policy and public health.

## Figures and Tables

**Table 1:** Mean daily dietary intake, by sex and age.

Nutrients	Men n = 779	Women n = 753	*p*-Value ^1^	55–65 Years n = 283	66–75 Years n = 570	>75 Years n = 679	*p*-Value ^2^
Macronutrient							
Energy (kcal)	1761 ± 517.9	1494 ± 432.9	<0.001	1744 ± 541.1 ^a^	1640 ± 498.0 ^b^	1573 ± 465.8 ^b^	<0.001
Protein (g)	70.9 ± 20.2	59.7 ± 17.0	<0.001	69.6 ± 20.7 ^a^	65.9 ± 19.3 ^b^	63.2 ± 18.8 ^c^	<0.001
Carbohydrate (g)	238.7 ± 74.4	210.3 ± 64.5	<0.001	234.7 ± 74.9 ^a^	227.3 ± 74.5 ^ab^	218.4 ± 65.9 ^b^	0.013
Fat (g)	64.9 ± 26.5	52.7 ± 20.6	<0.001	65.7 ± 28.4 ^a^	59.1 ± 23.8 ^b^	55.9 ± 23.0 ^c^	<0.001
SFA (g)	24.5 ± 10.6	20.0 ± 9.1	<0.001	24.5 ± 11.3 ^a^	22.1 ± 10.2 ^b^	21.5 ± 9.4 ^b^	<0.001
MUFA (g)	26.5 ± 12.1	21.1 ± 9.0	<0.001	27.1 ± 13.3 ^a^	24.0 ± 10.4 ^b^	22.4 ± 10.2 ^c^	<0.001
PUFA (g)	9.1 ± 4.6	7.6 ± 3.5	<0.001	9.3 ± 5.0 ^a^	8.7 ± 4.1 ^a^	7.8 ± 3.8 ^b^	<0.001
Dietary fiber (g)	18.8 ± 7.4	16.9 ± 6.0	<0.001	19.0 ± 7.0 ^a^	18.8 ± 7.2 ^a^	16.6 ± 6.2 ^b^	<0.001

Note: Data are presented as mean ± SD (standard deviation); MUFA—monounsaturated fatty acids; PUFA—polyunsaturated fatty acids; SFA—saturated fatty acids; ^1^ Mann–Whitney U test; ^2^ Kruskal–Wallis test; ^abc^ statistically significant differences between groups based on the NIR test.

**Table 2:** Mean daily dietary intake and adequacy of micronutrient intake by sex.

Nutrients	Sex	Age and Sex-Specific EAR	Dietary Intake	*p*-Value ^1^	D/SD_D_ > 1 ^2^	D/SD_D_ ≤1 and ≥−1 ^3^	D/SD_D_ < −1 ^4^	*p*-Value ^5^
			Mean ± SD		n (%)			
Vitamin A (µg)	M	630	1050 ± 1024	0.289	196 (25.2)	583 (74.8)	0 (0)	0.274
	W	500	997 ± 882		208 (27.6)	545 (72.4)	0 (0)	
Vitamin C (mg)	M	75	65.6 ± 54.1	0.019	107 (13.7)	366 (47.0)	306 (39.3)	<0.001
	W	60	69.6 ± 51.1		177 (23.5)	441 (58.6)	135 (17.9)	
Thiamine (mg)	M	1.1	1.13 ± 0.4	<0.001	178 (22.8)	412 (52.9)	189 (24.3)	0.046
	W	0.9	0.95 ± 0.3		172 (22.8)	436 (57.9)	145 (19.3)	
Riboflavin (mg)	M	1.1	1.48 ± 0.5	<0.001	369 (47.4)	369 (47.4)	41 (5.2)	0.002
	W	0.9	1.33 ± 0.5		393 (52.2)	344 (45.7)	16 (2.1)	
Niacin (mg)	M	12	16.27 ± 6.4	<0.001	361 (46.3)	384 (49.3)	34 (4.4)	0.001
	W	11	13.94 ± 5.5		279 (37.1)	431 (57.2)	43 (5.7)	
Vitamin B_6_ (mg)	M	1.4	1.65 ± 0.5	<0.001	312 (40.0)	383 (49.2)	84 (10.8)	0.048
	W	1.3	1.48 ± 0.5		256 (34.0)	410 (54.4)	87 (11.6)	
Vitamin B_12_ (µg)	M	2.0	4.45 ± 4.2	<0.001	211 (27.1)	568 (72.9)	0 (0)	0.816
	W	2.0	3.45 ± 3.2		200 (26.6)	553 (73.4)	0 (0)	
Folate (µg)	M	320	228.73 ± 83.4	<0.001	32 (4.1)	148 (19.0)	599 (76.9)	0.973
	W	320	211.76 ± 75.8		32 (4.2)	140 (18.6)	581 (77.2)	
Calcium (mg)	M	800	546.4 ± 266.2	0.010	32 (4.1)	98 (12.6)	649 (83.3)	<0.001
	W	1000	504.2 ± 222.5		5 (0.7)	53 (7.0)	695 (92.3)	
Phosphorus (mg)	M	580	1129.9 ± 364.6	<0.001	676 (86.8)	101 (13.0)	2 (0.2)	<0.001
	W	580	978.1 ± 309.0		545 (72.4)	202 (26.8)	6 (0.8)	
Magnesium (mg)	M	350	275.2 ± 90.2	<0.001	53 (6.8)	215 (27.6)	511 (65.6)	<0.001
	W	265	246.4 ± 76.2		113 (15.0)	394 (52.3)	246 (32.7)	
Zinc (mg)	M	9.4	9.4 ± 2.9	<0.001	166 (21.3)	402 (51.6)	211 (27.1)	<0.001
	W	6.8	7.8 ± 2.3		253 (33.6)	422 (56.0)	78 (10.4)	
Iron (mg)	M	6.0	10.6 ± 4.0	<0.001	569 (73.0)	207 (26.6)	3 (0.4)	<0.001
	W	6.0	9.0 ± 3.1		422 (56.0)	324 (43.0)	7 (0.9)	
Copper (mg)	M	0.7	1.1 ± 0.4	<0.001	513 (65.8)	257 (33.0)	9 (1.2)	<0.001
	W	0.7	1.0 ± 0.3		399 (53.0)	335 (44.5)	19 (2.5)	
Iodine (µg)	M	95	160.0 ± 52.8	<0.001	543 (69.7)	228 (29.3)	8 (1.0)	<0.001
	W	95	143.6 ± 46.9		449 (59.6)	290 (38.5)	14 (1.9)	

Note: M—men; W—women; EAR—Estimated Average Requirement; D—difference between observed individual intake and the EAR value; SD_D_—standard deviation of D value; ^1^ Mann–Whitney U test; ^2^ (D/SD_D_) > 1—it is a lot of confidence that the usual intake of a nutrient is adequate; ^3^ (D/SD_D_) ≤ 1 and ≥−1—it cannot be determined if the intake of an individual is adequate or inadequate; ^4^ (D/SD_D_) < −1—it is certain that the usual intake of a nutrient for the analyzed person is inadequate; ^5^ Chi-squared test.

**Table 3:** Mean daily dietary intake and adequacy of vitamin D and E intake by sex.

Nutrients	Sex	Reference Value (AI)	Dietary Intake	*p*-Value ^1^	Adequate	*p*-Value ^2^
			Mean ± SD		n (%)	
Vitamin D (µg)	M	15	3.24 ± 2.7	<0.001	5 (0.6)	0.275
	W	15	2.53 ± 2.5		2 (0.3)	
Vitamin E (mg)	M	10	7.64 ± 4.1	0.009	163 (20.9)	<0.001
	W	8	6.97 ± 3.3		245 (32.5)	

Note: M—men; W—women; AI—adequate intake; ^1^ Mann–Whitney U test ^2^ Chi-squared test.

**Table 4:** Mean daily dietary intake and adequacy of micronutrient intake by age.

Nutrients	Age	Dietary Intake	*p*-Value ^1^	D/SD_D_ > 1 ^2^	D/SD_D_ ≤1 and ≥−1 ^3^	D/SD_D_ < −1 ^4^	*p*-Value ^5^
		Mean ± SD		n (%)			
Vitamin A (µg)	55–65	1056 ± 953.4 ^a^		78 (27.6)	205 (72.4)	0 (0)	0.128
	66–75	1056 ± 949.1 ^a^	0.001	164 (28.8)	406 (71.2)	0 (0)	
	>75	983 ± 964.9 ^b^		162 (23.9)	517 (76.1)	0 (0)	
Vitamin C (mg)	55–65	74.8 ± 53.1 ^a^		64 (22.6)	159 (56.2)	60 (21.2)	<0.001
	66–75	70.5 ± 54.3 ^a^	<0.001	108 (18.9)	316 (55.4)	146 (25.6)	
	>75	62.2 ± 50.6 ^b^		112 (16.5)	332 (48.9)	235 (34.6)	
Thiamine (mg)	55–65	1.12 ± 0.4 ^a^		83 (29.3)	168 (59.4)	32 (11.3)	<0.001
	66–75	1.06 ± 0.4 ^b^	<0.00	137 (24.0)	320 (56.1)	113 (19.8)	
	>75	0.99 ± 0.4 ^c^		130 (19.1)	360 (53.0)	189 (27.8)	
Riboflavin (mg)	55–65	1.44 ± 0.5		156 (55.1)	123 (43.5)	4 (1.4)	0.044
	66–75	1.41 ± 0.5	0.085	285 (50.0)	265 (46.5)	20 (3.5)	
	>75	1.38 ± 0.5		321 (47.2)	325 (47.9)	33 (4.9)	
Niacin (mg)	55–65	16.41 ± 5.8 ^a^		147 (51.9)	132 (46.6)	4 (1.4)	<0.001
	66–75	15.61 ± 6.4 ^b^	<0.001	253 (44.4)	299 (52.4)	18 (3.2)	
	>75	14.19 ± 5.7 ^c^		240 (35.3)	384 (56.6)	55 (8.1)	
B_6_ (mg)	55–65	1.65 ± 0.5 ^a^		129 (45.6)	138 (48.8)	16 (5.6)	<0.001
	66–75	1.61 ± 0.5 ^a^	<0.001	224 (39.3)	299 (52.5)	47 (8.2)	
	>75	1.50 ± 0.5 ^b^		215 (31.7)	356 (52.4)	108 (15.9)	
B_12_ (µg)	55–65	3.90 ± 3.5		82 (29.0)	201 (71.0)	0 (0)	0.252
	66–75	3.98 ± 3.9	0.502	161 (28.2)	409 (71.8)	0 (0)	
	>75	3.96 ± 3.8		168 (24.7)	511 (75.3)	0 (0)	
Folate (µg)	55–65	235.52 ± 79.9 ^a^		22 (7.8)	53 (18.7)	208 (73.5)	0.009
	66–75	226.34 ± 82.0 ^b^	<0.001	22 (3.8)	115 (20.2)	433 (76.0)	
	>75	209.09 ± 77.2 ^c^		20 (2.9)	120 (17.7)	539 (79.4)	
Calcium (mg)	55–65	564.1 ± 269.3 ^a^		12 (4.2)	51 (18.0)	220 (77.7)	<0.001
	66–75	516.2 ± 235.5 ^b^	0.022	10 (1.8)	40 (7.0)	520 (91.2)	
	>75	517.6 ± 244.6 ^b^		15 (2.2)	60 (8.8)	604 (89.0)	
Phosphorus (mg)	55–65	1117.9 ± 39.9 ^a^		239 (84.4)	43 (15.2)	1 (0.4)	0.008
	66–75	1070.5 ± 64.3 ^b^	<0.001	469 (82.3)	99 (17.4)	2 (0.3)	
	>75	1016.4 ± 29.7 ^c^		513 (75.6)	161 (23.7)	5 (0.7)	
Magnesium (mg)	55–65	281.1 ± 84.2 ^a^		48 (17.0)	122 (43.1)	113 (39.9)	<0.001
	66–75	268.3 ± 91.4 ^b^	<0.001	67 (11.7)	246 (43.2)	257 (45.1)	
	>75	246.6 ± 76.6 ^c^		51 (7.5)	241 (35.5)	387 (57.0)	
Zinc (mg)	55–65	9.3 ± 2.9 ^a^		107 (37.8)	148 (52.3)	28 (9.9)	<0.001
	66–75	8.8 ± 2.9 ^b^	<0.001	162 (28.4)	312 (54.7)	96 (16.8)	
	>75	8.1 ± 2.5 ^c^		150 (22.1)	364 (53.6)	165 (24.3)	
Iron (mg)	55–65	10.5 ± 3.5 ^a^		221 (78.1)	61 (21.5)	1 (0.4)	<0.001
	66–75	10.1 ± 3.9 ^b^	<0.001	387 (67.9)	182 (31.9)	1 (0.2)	
	>75	9.2 ± 3.5 ^c^		383 (56.4)	288 (42.4)	8 (1.2)	
Copper (mg)	55–65	1.1 ± 0.3 ^a^		196 (69.3)	85 (30.0)	2 (0.7)	<0.001
	66–75	1.1 ± 0.4 ^a^	<0.001	357 (62.6)	208 (36.5)	5 (0.9)	
	>75	1.0 ± 0.3 ^b^		359 (52.9)	299 (44.0)	21 (3.1)	
Iodine (µg)	55–65	158.7 ± 55.6 ^a^		200 (70.7)	78 (27.5)	5 (1.8)	0.026
	66–75	154.1 ± 50.2 ^a^	0.011	378 (66.3)	187 (32.8)	5 (0.9)	
	>75	147.3 ± 48.5 ^b^		414 (61.0)	253 (37.2)	12 (1.8)	

Note: D—difference between observed individual intake and the EAR value; SD_D_—standard deviation of D value; ^1^ Kruskal–Wallis test; ^abc^ statistically significant differences between groups based on the NIR test; ^2^ (D/SD_D_) > 1—it is a lot of confidence that the usual intake of a nutrient is adequate; ^3^ (D/SD_D_) ≤ 1 and ≥−1—it cannot be determined if the intake of an individual is adequate or inadequate; ^4^ (D/SD_D_) < −1—it is certain that the usual intake of a nutrient for the analyzed person is inadequate; ^5^ Chi-squared test.

**Table 5:** Comparison of BRI values, age, energy intake, and micronutrients score by clusters.

Parameter	Cluster 1 n = 495	Cluster 2 n = 557	Cluster 3 n = 452	*p*-Value ^1^
BRI (Body Roundness Index)	4.97 ± 1.37 ^a^	5.34 ± 1.57 ^b^	7.08 ± 1.96 ^c^	<0.001
Age (years)	83.6 ± 7.5 ^a^	69.8 ± 9.7 ^b^	69.6 ± 8.4 ^b^	<0.001
Energy intake (kcal)	1414 ± 325 ^a^	2076 ± 441 ^b^	1322 ± 270 ^a^	<0.001
Micronutrients score (index)	7.3 ± 2.1 ^a^	11.5 ± 1.6 ^b^	7.5 ± 2.0 ^a^	<0.001

Note: Data are presented as mean ± SD (standard deviation); ^1^ ANOVA with adjustment by Bonferroni correction; ^abc^ different letters indicate statistically significant differences between clusters.

**Table 6:** Descriptive statistics (socio-demographic, physical activity level, and self-rated health status) of the study subgroups by clusters.

Parameter	Cluster 1		Cluster 2		Cluster 3		*p*-Value ^1^
	n	%	n	%	n	%	
Sex	n = 495		n = 557		n = 452		
women	222	44.8	245	44.0	269	59.5	<0.001
men	273	55.2	312	56.0	183	40.5	
Age group	n = 495		n = 557		n = 452		
55–65 years	3	0.6	172	30.9	107	18.8	<0.001
66–75 years	88	17.8	234	42.0	238	37.2	
>75 years	404	81.6	151	27.1	107	44.0	
Education	n = 492		n = 556		n = 451		
None/incomplete primary	94	19.1	17	3.1	22	4.9	<0.001
Primary	169	34.3	142	25.5	187	41.5	
Professional	175	35.6	273	49.1	194	43.0	
Secondary	10	2.0	27	4.9	8	1.8	
Higher	44	8.9	97	17.4	40	8.9	
Marital status	n = 493		n = 555		n = 451		
Widowed/divorced/ unmarried (single)	273	55.4	188	33.9	186	41.2	<0.001
Married; living with partner (not single)	220	44.6	367	66.1	265	58.8	
Self-rated health status (0—worst to 10—best)	n = 459		n = 546		n = 445		
0–3	41	8.9	19	3.5	31	7.0	<0.001
4–5	165	35.9	163	29.9	151	33.9	
6–7	149	32.5	206	37.7	166	37.3	
8–10	104	22.7	158	28.9	97	21.8	
Physical activity	n = 495		n = 557		n = 451		
Low	164	33.2	68	12.2	88	21.3	<0.001
Moderate	330	66.8	489	87.7	363	80.5	

Note: Data are presented as n (%). ^1^ Chi-squared test.

## Data Availability

The raw data supporting the conclusions of this article will be made available by the authors on request.

## Supplementary Materials

The following supporting information can be downloaded at: https://www.mdpi.com/article/10.3390/nu17233666/s1, Figure S1: Flow chart of the study population; Table S1: Participants’ age, anthropometric data, social and lifestyle variables; Table S2: Adequacy of micronutrient and anthropometric indices of men; Table S3: Adequacy of micronutrient and anthropometric indices of women; Table S4: BRI patterns by cluster analysis according to the input variables (Z-scores) among older adults.
