# Supplementary material for: Novel Anthropometric Indices and Probability of Adequate Nutrient Intake in the Older Polish Population
Source: Nutrients. 2025 Nov 24;17(23):3666. doi: 10.3390/nu17233666 (PMC12693771; doi:10.3390/nu17233666)
Supplement: Supplementary file 1 [file nutrients-17-03666-s001.zip › nutrients-3974507-supplementary.pdf]

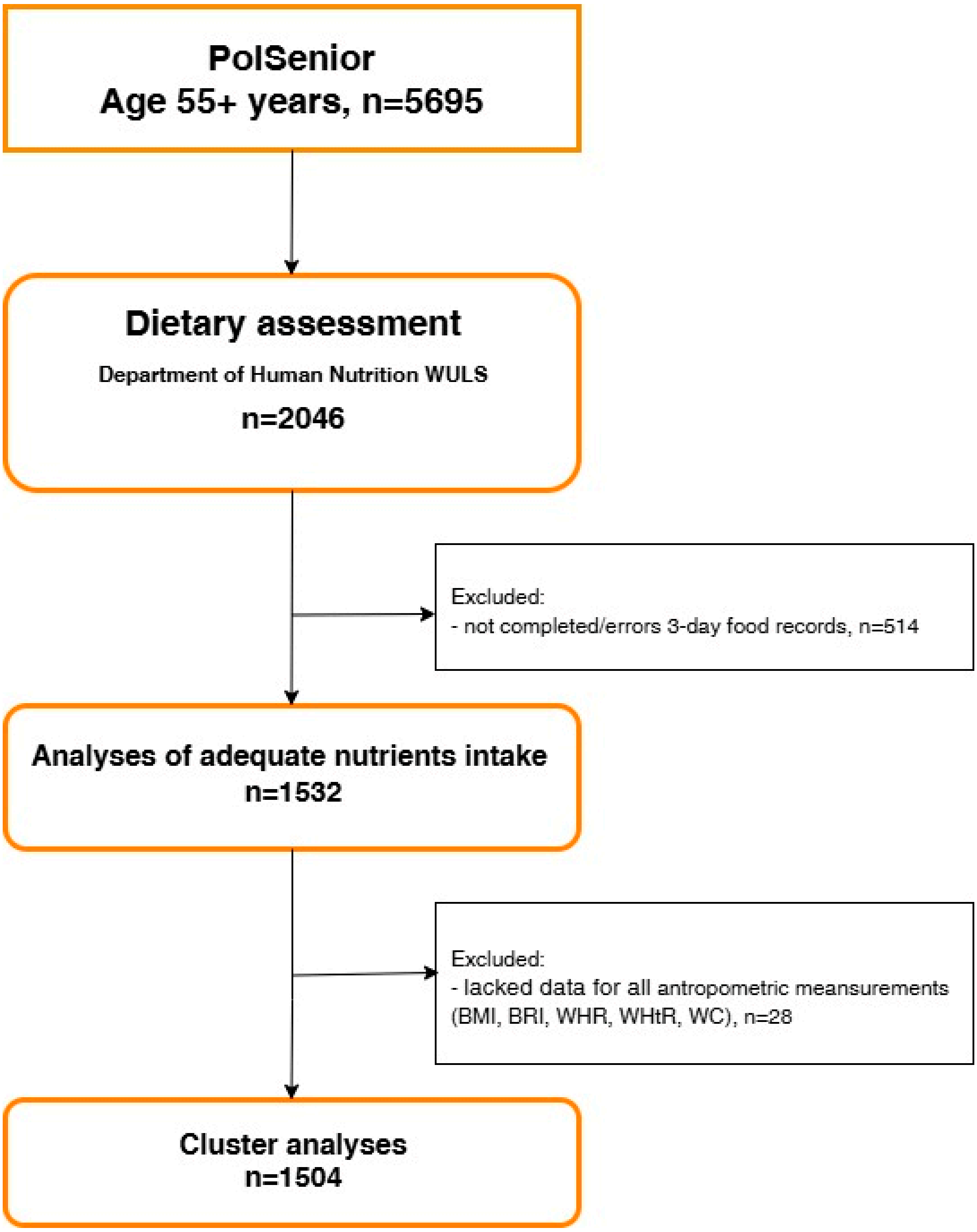

Figure S1. Flow chart of the study population.

Table S1. Participants' age, anthropometric data, social and lifestyle variables.

Characteristics	Men	Women	*p*-value[1]
	n (%)	n (%)	
Age (years)			
	n=779	n=753	
55-65	128 (16.4)	155 (20.6)	0.105
66-75	294 (37.8)	276 (36.6)	
>75	357 (45.8)	322 (42.8)	
BMI			
	n=765	n=738	
<23	106 (13.9)	93 (12.6)	< 0.001
23.0-29.9	449 (58.7)	364 (49.3)	
≥30	210 (27.4)	281 (38.1)	
BMI			
	n=765	n=738	
<24	155 (20.3)	131 (17.8)	< 0.001
24.0-28.9	331 (43.3)	266 (36.0)	
≥29	279 (36.5)	341 (46.2)	
WHR ratio			
	n=768	n=736	
<0.9 for men; <0.85 for women	130 (16.9)	250 (34.0)	1.000
≥0.9 for men; ≥0.85 for women	638 (83.1)	486 (66.0)	
WHtR ratio			
	n=768	n=737	
<0.5	45 (5.9)	56 (7.6)	0.178
≥0.5	723 (94.1)	681 (92.4)	
Education			
	n=777	n=750	
None/incomplete primary	55 (7.1)	86 (11.5)	
Primary	232 (29.9)	279 (37.2)	< 0.001
Professional	160 (20.6)	80 (10.7)	
Secondary	204 (26.2)	203 (27.0)	
Higher	126 (16.2)	102 (13.6)	
Marital status			
	n=776	n=750	
Unmarried	15 (1.9)	27 (3.6)	< 0.001
Married	587 (75.4)	274 (36.4)	
Divorced	11 (1.4)	27 (3.6)	
Widowed	163 (20.9)	422 (56.0)	
Self-rated health status (numerical scale: 0 – worst to 10 – best)			
	n=722	n=747	
0-3	45 (6.0)	51 (7.1)	0.332
4-5	235 (31.5)	251 (34.8)	
6-7	271 (36.3)	253 (35.0)	
8-10	196 (26.2)	167 (23.1)	
Physical activity			
	n=778	n=752	
Moderate	638 (82.0)	552 (73.4)	< 0.001
Low	140 (18.0)	200 (26.6)	
Alcohol intake			
	n=772	n=744	
Yes	628 (81.3)	447 (60.1)	< 0.001
No	144 (18.7)	297 (39.9)	

Smoking (current or in the past)	n=777	n=750	< 0.001
Yes	537 (69.1)	194 (25.9)	
No	240 (30.9)	556 (74.1)	

Note: body mass index (BMI), waist-hip ratio (WHR), waist-to-height ratio (WHtR);

[1] Significance is indicated by $p < 0.05$, chi-squared test.

Table S2. Adequacy of micronutrient and anthropometric indices of men.

Nutrients	Adequacy of micronutrient	BRI	BMI [kg/m²]	WC [cm]	WHR	WHtR
Vitamin C (mg)	$D/SD_D > 1$[1]	5.5 ± 1.6	29.2 ± 10.7	101.8 ± 10.9	0.962 ± 0.078	0.601 ± 0.068
	$D/SD_D \leq 1$ and ≥ -1[2]	5.6 ± 1.6	29.4 ± 10.9	102.4 ± 11.5	0.968 ± 0.078	0.605 ± 0.068
	$D/SD_D < -1$[3]	5.4 ± 1.7	28.5 ± 10.5	100.1 ± 12.7	0.964 ± 0.079	0.595 ± 0.076
Thiamine (mg)	$D/SD_D > 1$[1]	5.6 ± 1.6	29.0 ± 10.4	102.4 ± 11.7	0,970 ± 0.081	0,605 ± 0.068
	$D/SD_D \leq 1$ and ≥ -1[2]	5.5 ± 1.6	28.8 ± 9.7	101.5 ± 11.4	0,967 ± 0.082	0,601 ± 0.069
	$D/SD_D < -1$[3]	5.5 ± 1.8	29.3 ± 12.9	100.4 ± 13.2	0,958 ± 0.068	0,596 ± 0.079
Riboflavin (mg)	$D/SD_D > 1$[1]	5.5 ± 1.6	28.8 ± 10.2	101.7 ± 11.6	0.965 ± 0.079	0.601 ± 0.068
	$D/SD_D \leq 1$ and ≥ -1[2]	5.6 ± 1.7	29.2 ± 11.0	101.5 ± 2.1	0.968 ± 0.079	0.601 ± 0.073
	$D/SD_D < -1$[3]	5.3 ± 1.9	28.5 ± 12.8	98.6 ± 14.2	0.952 ± 0.069	0.589 ± 0.085
Niacin (mg)	$D/SD_D > 1$[1]	5.5 ± 1.5	28.6 ± 8.7	101.7 ± 11.1	0.966 ± 0.078	0.601 ± 0.065
	$D/SD_D \leq 1$ and ≥ -1[2]	5.6 ± 1.7	29.3 ± 12.0	101.4 ± 12.3	0.967 ± 0.079	0.601 ± 0.074
	$D/SD_D < -1$[3]	5.3 ± 2.1	29.9 ± 13.9	98.6 ± 15.7	0.940 ± 0.069	0.587 ± 0.094
B6 (mg)	$D/SD_D > 1$[1]	5.5 ± 1.5	28.9 ± 10.1	101.5 ± 11.2	0.964 ± 0.082	0.598 ± 0.066
	$D/SD_D \leq 1$ and ≥ -1[2]	5.6 ± 1.7	29.0 ± 10.1	101.7 ± 12.2	0.968 ± 0.070	0.603 ± 0.074
	$D/SD_D < -1$[3]	5.5 ± 1.8	29.5 ± 14.8	100.1 ± 13.6	0.962 ± 0.098	0.596 ± 0.080
Folate (mcg)	$D/SD_D > 1$[1]	5.2 ± 1.1	28.0 ± 3.8	100.4 ± 8.2	0.960 ± 0.050	0.587 ± 0.049
	$D/SD_D \leq 1$ and ≥ -1[2]	5.5 ± 1.6	28.2 ± 7.3	101.8 ± 11.6	0.966 ± 0.084	0.600 ± 0.067
	$D/SD_D < -1$[3]	5.6 ± 1.7	29.2 ± 11.6	101.4 ± 12.2	0.966 ± 0.078	0.601 ± 0.073
Calcium (mg)	$D/SD_D > 1$[1]	5.6 ± 1.6	30.5 ± 13.6	102.4 ± 10.9	0.977 ± 0.054	0.602 ± 0.064
	$D/SD_D \leq 1$ and ≥ -1[2]	5.2 ± 1.5	27.8 ± 8.6	100.2 ± 11.7	0.963 ± 0.084	0.588 ± 0.067
	$D/SD_D < -1$[3]	5.6 ± 1.7	29.1 ± 10.8	101.6 ± 12.1	0.966 ± 0.078	0.602 ± 0.072
Magnesium (mg)	$D/SD_D > 1$[1]	5.3 ± 1.5	28.9 ± 10.9	100.8 ± 9.7	0.959 ± 0.099	0.590 ± 0.060
	$D/SD_D \leq 1$ and ≥ -1[2]	5.6 ± 1.6	28.7 ± 8.2	102.2 ± 12.0	0.969 ± 0.071	0.605 ± 0.069
	$D/SD_D < -1$[3]	5.5 ± 1.7	29.1 ± 11.6	101.2 ± 12.2	0.965 ± 0.079	0.600 ± 0.073
Zinc (mg)	$D/SD_D > 1$[1]	5.7 ± 1.8	29.3 ± 10.7	102.9 ± 12.7	0.971 ± 0.085	0.605 ± 0.075
	$D/SD_D \leq 1$ and ≥ -1[2]	5.6 ± 1.6	29.1 ± 10.5	101.6 ± 11.3	0.964 ± 0.071	0.602 ± 0.068
	$D/SD_D < -1$[3]	5.4 ± 1.7	28.6 ± 11.1	99.9 ± 12.5	0.963 ± 0.086	0.594 ± 0.075
Iron (mg)	$D/SD_D > 1$[1]	5.5 ± 1.6[a]	28.9 ± 10.1[a]	101.5 ± 11.7[a]	0.965 ± 0.076	0.600 ± 0.070[a]
	$D/SD_D \leq 1$ and ≥ -1[2]	5.6 ± 1.7[a]	29.3 ± 12.2[a]	101.6 ± 12.5[a]	0.969 ± 0.082	0.605 ± 0.073[a]
	$D/SD_D < -1$[3]	3.0 ± 1.6[b]	20.6 ± 2.8[b]	79.2 ± 13.0[b]	0.831 ± 0.102	0.474 ± 0.087[b]
	$D/SD_D > 1$[1]	5.5 ± 1.6	28.9 ± 10.0	101.4 ± 11.7	0.963 ± 0.075	0.600 ± 0.070

Copper (mg)	D/SD_D ≤1 and ≥-1[2]	5.6 ± 1.7	29.0 ± 11.2	101.7 ± 12.3	0.973 ± 0.083	0.602 ± 0.072
	D/SD_D < -1[3]	4.8 ± 2.0	33.5 ± 25.8	93.8 ± 15.0	0.929 ± 0.104	0.566 ± 0.097
Iodine (mcg)	D/SD_D > 1[1]	5.5 ± 1.6	28.9 ± 10.3	101.6 ± 11.9	0.965 ± 0.078	0.601 ± 0.070
	D/SD_D ≤1 and ≥-1[2]	5.6 ± 1.7	29.3 ± 11.7	101.3 ± 11.8	0.968 ± 0.078	0.602 ± 0.073
	D/SD_D < -1[3]	4.4 ± 1.6	24.8 ± 4.9	93.4 ± 15.9	0.925 ± 0.100	0.546 ± 0.083

Note:

[1] (D/SD_D) > 1 - it is a lot of confidence that the usual intake of a nutrient is adequate;

[2] (D/SD_D) ≤1 and ≥-1, - it cannot be determined if the intake of an individual is adequate or inadequate;

[3] (D/SD_D) < -1 - it is certain that the usual intake of a nutrient for the analyzed person is inadequate;

[abc] statistically significant differences between groups based on the NIR test ($p < 0.05$; Kruskal-Wallis test)

Table S3. Adequacy of micronutrient and anthropometric indices of women.

Nutrients	Adequacy of micronutrient	BRI	BMI [kg/m^2]	WC [cm]	WHR	WHtR
Vitamin C (mg)	$D/SD_D > 1$[1]	5.9 ± 1.9	32.2 ± 14.0[a]	96.7 ± 12.0	0.872 ± 0.088[a]	0.616 ± 0.079
	$D/SD_D \leq 1$ and ≥ -1[2]	5.9 ± 2.2	30.0 ± 10.0[b]	96.3 ± 13.8	0.875 ± 0.080[a]	0.615 ± 0.091
	$D/SD_D < -1$[3]	6.1 ± 1.9	29.5 ± 11.9[b]	97.0 ± 13.0	0.895 ± 0.076[b]	0.623 ± 0.079
Thiamine (mg)	$D/SD_D > 1$[1]	5.9 ± 1.9	30.8 ± 11.9	96.1 ± 12.5	0.870 ± 0.077	0.613 ± 0.082
	$D/SD_D \leq 1$ and ≥ -1[2]	6.0 ± 2.2	30.4 ± 11.7	96.7 ± 13.7	0.881 ± 0.088	0.617 ± 0.090
	$D/SD_D < -1$[3]	6.0 ± 1.9	30.0 ± 10.1	96.6 ± 12.6	0.879 ± 0.067	0.617 ± 0.081
Riboflavin (mg)	$D/SD_D > 1$[1]	5.8 ± 2.0	31.1 ± 13.5	95.8 ± 13.0	0.877 ± 0.092	0.612 ± 0.086
	$D/SD_D \leq 1$ and ≥ -1[2]	6.0 ± 2.1	29.7 ± 8.7	97.3 ± 13.5	0.878 ± 0.070	0.620 ± 0.087
	$D/SD_D < -1$[3]	6.4 ± 1.9	28.2 ± 6.3	97.2 ± 13.8	0.891 ± 0.047	0.637 ± 0.076
Niacin (mg)	$D/SD_D > 1$[1]	6.0 ± 2.1	30.3 ± 9.2	96.7 ± 13.0	0.872 ± 0.083	0.618 ± 0.087
	$D/SD_D \leq 1$ and ≥ -1[2]	5.9 ± 2.0	30.4 ± 12.2	96.4 ± 13.5	0.880 ± 0.082	0.614 ± 0.087
	$D/SD_D < -1$[3]	6.2 ± 2.0	31.2 ± 16.4	96.4 ± 12.1	0.894 ± 0.065	0.627 ± 0.081
B6 (mg)	$D/SD_D > 1$[1]	6.0 ± 2.0	30.5 ± 10.3	96.5 ± 12.8	0.873 ± 0.083	0.617 ± 0.085
	$D/SD_D \leq 1$ and ≥ -1[2]	5.9 ± 2.1	30.4 ± 12.0	96.4 ± 13.6	0.879 ± 0.083	0.615 ± 0.089
	$D/SD_D < -1$[3]	6.1 ± 2.0	30.4 ± 12.2	96.8 ± 12.7	0.886 ± 0.074	0.622 ± 0.082
Folate (mcg)	$D/SD_D > 1$[1]	5.3 ± 2.3[a]	28.6 ± 5.7	93.8 ± 14.0	0.862 ± 0.081[a]	0.588 ± 0.091[a]
	$D/SD_D \leq 1$ and ≥ -1[2]	5.8 ± 2.2[ab]	30.6 ± 12.1	95.3 ± 14.9	0.870 ± 0.087[ab]	0.607 ± 0.093[ab]
	$D/SD_D < -1$[3]	6.0 ± 2.0[b]	30.5 ± 11.5	97.0 ± 12.7	0.880 ± 0.080[b]	0.620 ± 0.084[b]
Calcium (mg)	$D/SD_D > 1$[1]	5.6 ± 1.1	25.9 ± 3.0	91.1 ± 8.20	0.878 ± 0.067	0.604 ± 0.048
	$D/SD_D \leq 1$ and ≥ -1[2]	5.6 ± 2.3	31.9 ± 18.0	93.5 ± 13.7	0.871 ± 0.082	0.598 ± 0.097
	$D/SD_D < -1$[3]	6.0 ± 2.0	30.3 ± 10.8	96.8 ± 13.2	0.878 ± 0.082	0.618 ± 0.086
Magnesium (mg)	$D/SD_D > 1$[1]	5.5 ± 2.0[a]	29.6 ± 8.6	93.9 ± 13.2	0.859 ± 0.085[a]	0.598 ± 0.087[a]
	$D/SD_D \leq 1$ and ≥ -1[2]	6.0 ± 2.1[ab]	30.4 ± 11.6	96.6 ± 13.4	0.877 ± 0.085[b]	0.616 ± 0.088[b]
	$D/SD_D < -1$[3]	6.1 ± 2.0[b]	30.7 ± 12.3	97.6 ± 18.8	0.888 ± 0.070[b]	0.625 ± 0.082[b]
Zinc (mg)	$D/SD_D > 1$[1]	6.0 ± 2.0	31.5 ± 12.8[a]	96.7 ± 12.5	0.869 ± 0.075	0.618 ± 0.083
	$D/SD_D \leq 1$ and ≥ -1[2]	5.9 ± 2.1	29.9 ± 10.8[b]	96.4 ± 13.9	0.881 ± 0.089	0.614 ± 0.090
	$D/SD_D < -1$[3]	6.1 ± 1.9	29.3 ± 9.8[b]	96.6 ± 11.4	0.888 ± 0.056	0.624 ± 0.079
Iron (mg)	$D/SD_D > 1$[1]	5.9 ± 2.1	31.2 ± 13.1	96.3 ± 13.3	0.871 ± 0.082[a]	0.615 ± 0.088
	$D/SD_D \leq 1$ and ≥ -1[2]	6.0 ± 2.0	29.2 ± 7.8	96.8 ± 13.2	0.886 ± 0.081[b]	0.618 ± 0.085
	$D/SD_D < -1$[3]	6.0 ± 1.4	37.2 ± 28.4	94.8 ± 11.7	0.901 ± 0.043[b]	0.624 ± 0.055
Copper (mg)	$D/SD_D > 1$[1]	5.9 ± 2.0	30.7 ± 11.9	96.2 ± 13.0	0.874 ± 0.086	0.614 ± 0.086

	D/SD$_D$ ≤1 and ≥-1[2]	6.0 ± 2.1	30.1 ± 10.4	97.2 ± 13.5	0.882 ± 0.077	0.620 ± 0.088
	D/SD$_D$ < -1[3]	5.9 ± 1.9	30.6 ± 18.0	92.7 ± 11.6	0.890 ± 0.061	0.614 ± 0.080
Iodine (mcg)	D/SD$_D$ > 1[1]	5.8 ± 2.0	30.2 ± 10.8	96.0 ± 12.8	0.876 ± 0.087	0.612 ± 0.085
	D/SD$_D$ ≤1 and ≥-1[2]	6.1 ± 2.1	30.7 ± 12.5	97.2 ± 13.8	0.880 ± 0.073	0.622 ± 0.089
	D/SD$_D$ < -1[3]	6.5 ± 2.0	29.8 ± 5.7	99.3 ± 13.8	0.884 ± 0.056	0.641 ± 0.087

Note:

[1] (D/SD$_D$) > 1 - it is a lot of confidence that the usual intake of a nutrient is adequate;

[2] (D/SD$_D$) ≤1 and ≥-1, - it cannot be determined if the intake of an individual is adequate or inadequate;

[3] (D/SD$_D$) < -1 - it is certain that the usual intake of a nutrient for the analyzed person is inadequate;

[abc] statistically significant differences between groups based on the NIR test ($p < 0.05$; Kruskal-Wallis test)

Table S4. BRI patterns by cluster analysis according to the input variables (Z-scores) among older adults.

Parameter	Cluster 1 n=495	Cluster 2 n=557	Cluster 3 n=452	*p*-value[1]
BRI (Body Roundness Index) (z-score)	-0.41 ± 0.73[a]	-0.21 ± 0.84[b]	0.72 ± 1.05[c]	< 0.001
Age, years (z-score)	0.86 ± 0.69[a]	-0.41 ± 0.88[b]	-0.43 ± 0.78[b]	< 0.001
Energy intake, kcal (z-score)	-0.44 ± 0.66[a]	0.90 ± 0.89[b]	-0.62 ± 0.45[a]	< 0.001
Micronutrients score (z-score)	-0.58 ± 0.77[a]	0.93 ± 0.59[b]	-0.51 ± 0.75[a]	< 0.001

Note: Data are presented as n (%), mean (SD).

[1] Different letters indicate significant differences between clusters ($p < 0.05$; Benferroni post-hoc test)
